# Supplementary material for: Intracortical recordings reveal vision-to-action cortical gradients driving human exogenous attention
Source: Nat Commun. 2024 Mar 26;15:2586. doi: 10.1038/s41467-024-46013-4 (PMC10965949; doi:10.1038/s41467-024-46013-4)
Supplement: Supplementary file 3 — Description of Additional Supplementary Files [file 41467_2024_46013_MOESM3_ESM.pdf]

Description of Additional Supplementary Files

File name: Supplementary Movie 1

Description: Localization of target-locked clusters. Brain visualization was done using BrainNet Viewer Matlab toolbox (Xia M, Wang J, He Y (2013) BrainNet Viewer: A Network Visualization Tool for Human Brain Connectomics. PLoS ONE 8(7): e68910. doi:10.1371/journal.pone.0068910).

File name: Supplementary Movie 2

Description: Localization of response-locked clusters. Brain visualization was done using BrainNet Viewer Matlab toolbox (Xia M, Wang J, He Y (2013) BrainNet Viewer: A Network Visualization Tool for Human Brain Connectomics. PLoS ONE 8(7): e68910. doi:10.1371/journal.pone.0068910).
