# Supplementary material for: Intracortical recordings reveal vision-to-action cortical gradients driving human exogenous attention
Source: Nat Commun. 2024 Mar 26;15:2586. doi: 10.1038/s41467-024-46013-4 (PMC10965949; doi:10.1038/s41467-024-46013-4)
Supplement: Supplementary file 6 — Reporting Summary [file 41467_2024_46013_MOESM6_ESM.pdf]

nature portfolio

Corresponding author(s): Tal SEIDEL MALKINSON

Last updated by author(s): 26/01/2024

Reporting Summary

Nature Portfolio wishes to improve the reproducibility of the work that we publish. This form provides structure for consistency and transparency in reporting. For further information on Nature Portfolio policies, see our Editorial Policies and the Editorial Policy Checklist.

Statistics

For all statistical analyses, confirm that the following items are present in the figure legend, table legend, main text, or Methods section.

n/a	Confirmed	
☐	☒	The exact sample size (*n*) for each experimental group/condition, given as a discrete number and unit of measurement
☐	☒	A statement on whether measurements were taken from distinct samples or whether the same sample was measured repeatedly
☐	☒	The statistical test(s) used AND whether they are one- or two-sided<br>*Only common tests should be described solely by name; describe more complex techniques in the Methods section.*
☐	☒	A description of all covariates tested
☐	☒	A description of any assumptions or corrections, such as tests of normality and adjustment for multiple comparisons
☐	☒	A full description of the statistical parameters including central tendency (e.g. means) or other basic estimates (e.g. regression coefficient) AND variation (e.g. standard deviation) or associated estimates of uncertainty (e.g. confidence intervals)
☐	☒	For null hypothesis testing, the test statistic (e.g. *F*, *t*, *r*) with confidence intervals, effect sizes, degrees of freedom and *P* value noted<br>*Give P values as exact values whenever suitable.*
☐	☒	For Bayesian analysis, information on the choice of priors and Markov chain Monte Carlo settings
☒	☐	For hierarchical and complex designs, identification of the appropriate level for tests and full reporting of outcomes
☐	☒	Estimates of effect sizes (e.g. Cohen's *d*, Pearson's *r*), indicating how they were calculated

Our web collection on statistics for biologists contains articles on many of the points above.

Software and code

Policy information about availability of computer code

Data collection	E-prime 3.0 software (Psychology Software Tools, Pittsburgh, PA) controlled the presentation of stimuli, timing operations, and data collection.
Data analysis	Data preprocessing was done using FieldTrip toolbox for EEG/MEG-analysis (Donders Institute for Brain, Cognition and Behaviour, Radboud University, the Netherlands. See http://fieldtriptoolbox.org 91) and Matlab (Matlab R2016b and R2020a, The MathWorks, Inc.), and JASP (Version 0.14.1). Visualisation of brain localization of sEEG contacts in standard MNI152 space was done using the the BrainNet Viewer matlab toolbox (http://www.nitrc.org/projects/bnv/ bnv/). The structural connectivity preprocessing and analysis was done using TrackVis (0.6.1 http://trackvis.org/), FSL (6.0 https://fsl.fmrib.ox.ac.uk/fsl/fslwiki/FSL) and BCBtoolkit (http://toolkit.bcblab.com/).

For manuscripts utilizing custom algorithms or software that are central to the research but not yet described in published literature, software must be made available to editors and reviewers. We strongly encourage code deposition in a community repository (e.g. GitHub). See the Nature Portfolio guidelines for submitting code & software for further information.

Data

Policy information about availability of data

All manuscripts must include a data availability statement. This statement should provide the following information, where applicable:

- Accession codes, unique identifiers, or web links for publicly available datasets
- A description of any restrictions on data availability
- For clinical datasets or third party data, please ensure that the statement adheres to our policy

Raw data cannot be shared due to ethics committee restrictions. Intermediate as well as final processed data that support the findings of this study are available from the corresponding author (T.S.M.) upon request. The diffusion MRI data used in this study are available in the HCP database https://www.humanconnectome.org/study/hcp-young-adult/document/1200-subjects-data-release

Research involving human participants, their data, or biological material

Policy information about studies with human participants or human data. See also policy information about sex, gender (identity/presentation), and sexual orientation and race, ethnicity and racism.

Reporting on sex and gender	16 women and 15 men participated in the study. Gender is reported based on the participants' clinical records. Gender and sex were not used in the analysis as the data of all participating patients were pooled in the analysis and the individual electrode implantation scheme of each patient does not allow a breakdown.
Reporting on race, ethnicity, or other socially relevant groupings	It is illegal in France (article 6 of Law n° 78-17 of January 6th 1978 " Informatique et libertés ") to collect information on race and ethnicity.
Population characteristics	Patients were aged 31.8 ± 8.3 years, Table 1 details their handedness.
Recruitment	The recruitment was carried out in the Epilepsy service of Pitié-Salpêtrière Hospital in Paris, independently of the research protocol, based solely on medical criteria for epilepsy treatment. We included in the study all epileptic patients implanted with electrodes as part of their medical treatment who gave their consent to participate. Patients were explicitly informed that their participation in the study was entirely separate from their medical treatment, had no bearing on it, and they could choose to withdraw without any impact on their treatment. Since recruitment was independent of the study, any potential self-selection bias is more relevant to the medical intervention and not to the research or its results.
Ethics oversight	CPP Paris VI, Pitié-Salpêtrière Hospital, INSERM C11-16

Note that full information on the approval of the study protocol must also be provided in the manuscript.

Field-specific reporting

Please select the one below that is the best fit for your research. If you are not sure, read the appropriate sections before making your selection.

☐ Life sciences ☒ Behavioural & social sciences ☐ Ecological, evolutionary & environmental sciences

For a reference copy of the document with all sections, see nature.com/documents/nr-reporting-summary-flat.pdf

Behavioural & social sciences study design

All studies must disclose on these points even when the disclosure is negative.

Study description	Quantitative data acquired through intracerebral recordings in epileptic patients and behavioral measures (RT) were used to investigate the cortical correlates of exogenous visual attention using a Posner attention task. The task manipulated the location and timing of the presentation of visual stimuli evoking attention exogenous capturing. The spatiotemporal dynamics of neural responses across intracerebral contacts were analysed and their relationship to individual task performance was explored. Electrode localization in patients' brains was based on clinically acquired CT and structural MRI images.
Research sample	Thirty one patients (aged 31.8 ± 8.3 years, 16 women; See Table 1 for full details) with drug-resistant focal epilepsy, hospitalized at the Pitié-Salpêtrière Hospital in Paris. Structural connectivity analysis was performed using 176 healthy subjects from the HCP young adults 7T diffusion MRI dataset (https://www.humanconnectome.org/study/hcp-young-adult/document/1200-subjects-data-release).
Sampling strategy	We included 100% the patients that underwent the medical procedure of electrode implantation for treating their refractory epilepsy and gave their consent for participating in our study, over the course of 8.5 years. Since data collection is extremely challenging, we aimed at having a sample of N=30.
Data collection	For medical reasons, patients underwent intracerebral recordings using stereotactically implanted multi-wire intracerebral depth electrodes (iEEG). The experimental recordings took place 4-14 days after implantation, during which their antiepileptic medication

was gradually tapered and/or discontinued. The patients were implanted with 5-12 platinum electrodes (AdTech®, Wisconsin), each having 4-12 contacts, measuring 1.12 mm in diameter and 2.41 mm in length, with nickel-chromium wiring. The distance between the centers of two contacts was set at 5 mm. Electrode placement was determined based on clear clinical criteria.

In 13 patients, neural recordings were conducted using an audio-video EEG monitoring system (Micromed), enabling the simultaneous recording of 128 depth EEG channels sampled at 1024 Hz (0.18 to 220 Hz bandwidth). In 18 patients, recordings were performed with a Neuralynx system (ATLAS, Neuralynx, Inc.), allowing for the recording of up to 160 depth EEG channels sampled at 4 KHz (0.1 to 1000 Hz bandwidth).

A Dell Latitude D600 PC running E-prime 3.0 software (Psychology Software Tools, Pittsburgh, PA) controlled stimulus presentation, timing operations, and data collection. During the experiment, patients were seated approximately 57 cm away from the computer screen in their hospital room. Only the researcher was present with the patient during the experiment, verifying their gaze position by confrontation. Although the researcher was not blinded to the experimental condition and/or the study hypothesis, the acquisition software delivered experimental conditions randomly, ensuring that the researcher was blinded to the order of the presented experimental conditions.

Timing

The data were collected between 14/03/2012 and 13/09/2019, paced by clinical admissions that were determined independently of any experimental considerations.

Data exclusions

Three patients were excluded post hoc because of severe cognitive impairments and abnormally long response times (1 patient) or because of the presence of wide-spread brain lesions (2 patients).

Non-participation

No participants dropped out/declined participation.

Randomization

No randomization was done as data were collected in epileptic patients implanted with electrodes in the course of their medical treatment.

Reporting for specific materials, systems and methods

We require information from authors about some types of materials, experimental systems and methods used in many studies. Here, indicate whether each material, system or method listed is relevant to your study. If you are not sure if a list item applies to your research, read the appropriate section before selecting a response.

Materials & experimental systems

n/a	Involved in the study
☒	☐ Antibodies
☒	☐ Eukaryotic cell lines
☒	☐ Palaeontology and archaeology
☒	☐ Animals and other organisms
☒	☐ Clinical data
☒	☐ Dual use research of concern
☒	☐ Plants

Methods

n/a	Involved in the study
☒	☐ ChIP-seq
☒	☐ Flow cytometry
☐	☒ MRI-based neuroimaging

Magnetic resonance imaging

Experimental design

Design type

Anatomical T1 images acquired clinically for electrodes localization in the implanted patients in the course of their medical treatment

Design specifications

no experimental design

Behavioral performance measures

no behavioural measures during MRI T1 images acquisition

Acquisition

Imaging type(s)

structural

Field strength

1.5T or 3T

Sequence & imaging parameters

T1 Mprage 1mm iso

Area of acquisition

whole brain

Diffusion MRI ☐ Used ☒ Not used

Preprocessing

Preprocessing software	Spatial localization of the electrode was automatically computed in native space using the Epiloc toolbox developed by the STIM engineering facility at the Paris Brain Institute (https://icm-institute.org/fen/cenir-stim//) also using Freesurfer image analysis suite (http://surfer.nmr.mgh.harvard.edu/) that is embedded in Epiloc.
Normalization	data were not normalized for electrode localization. They were only normalized for visualization purposes
Normalization template	For visualization of electrode localization across patients data were normalized using the MNI152 template provided in Freesurfer image analysis suite (http://surfer.nmr.mgh.harvard.edu/)
Noise and artifact removal	No noise or artifact removal iwere peformed on the structural MRI data
Volume censoring	NA

Statistical modeling & inference

Model type and settings	NA
Effect(s) tested	NA

Specify type of analysis: ☒ Whole brain ☐ ROI-based ☐ Both

Statistic type for inference (See Eklund et al. 2016)	NA
Correction	NA

Models & analysis

n/a	Involved in the study
☒	☐ Functional and/or effective connectivity
☒	☐ Graph analysis
☒	☐ Multivariate modeling or predictive analysis
